# Asymmetric Dimethylarginine, Endothelial Dysfunction and Renal Disease

**DOI:** 10.3390/ijms130911288

**Published:** 2012-09-10

**Authors:** Luis Aldámiz-Echevarría, Fernando Andrade

**Affiliations:** Division of Metabolism, Cruces University Hospital, Barakaldo, Basque Country 48903, Spain; E-Mail: fernando.andradelodeiro@osakidetza.net

**Keywords:** asymmetric dimethylarginine (ADMA), arginine (Arg), children, dimethylarginine dimethylaminohydrolase (DDAH), endothelial dysfunction, kidney, methylarginines, nitric oxide, oxidative stress, renal failure

## Abstract

l-Arginine (Arg) is oxidized to l-citrulline and nitric oxide (NO) by the action of endothelial nitric oxide synthase (NOS). In contrast, protein-incorporated Arg residues can be methylated with subsequent proteolysis giving rise to methylarginine compounds, such as asymmetric dimethylarginine (ADMA) that competes with Arg for binding to NOS. Most ADMA is degraded by dimethylarginine dimethyaminohydrolase (DDAH), distributed widely throughout the body and regulates ADMA levels and, therefore, NO synthesis. In recent years, several studies have suggested that increased ADMA levels are a marker of atherosclerotic change, and can be used to assess cardiovascular risk, consistent with ADMA being predominantly absorbed by endothelial cells. NO is an important messenger molecule involved in numerous biological processes, and its activity is essential to understand both pathogenic and therapeutic mechanisms in kidney disease and renal transplantation. NO production is reduced in renal patients because of their elevated ADMA levels with associated reduced DDAH activity. These factors contribute to endothelial dysfunction, oxidative stress and the progression of renal damage, but there are treatments that may effectively reduce ADMA levels in patients with kidney disease. Available data on ADMA levels in controls and renal patients, both in adults and children, also are summarized in this review.

## 1. Introduction

In addition to the traditional cardiovascular risk factors, other factors associated with renal failure involved in common metabolic pathways have recently been studied in detail. The metabolism of amino acids tends to be impaired in patients with chronic renal failure due to abnormalities in their synthesis or excretion. For example, one of the alterations of amino acid metabolism in patients with chronic renal failure is hyperhomocysteinaemia; this is considered to be an independent risk factor for cardiovascular disease [1] and occurs in more than 85% of patients after kidney transplant (RTx) and in those with end-stage renal failure [2].

The new metabolic pathways currently being described may help, to some extent, to group these conditions by pathophysiological characteristics and to identify possible alternative therapeutic approaches. In particular, it seems interesting to study the metabolism of arginine and associated metabolic pathways. For this, it is useful to determine the levels of arginine and its methylated derivatives as part of the analysis of the cardiovascular risk and endothelial dysfunction of renal disease.

## 2. Nitric Oxide and the Kidney

**Figure 1:**
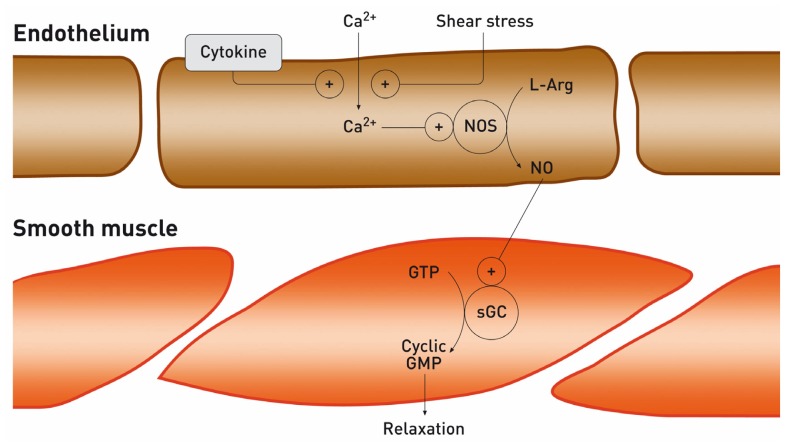
Nitric oxide (NO) synthesis in vascular endothelium and its diffusion to smooth-muscle cells where soluble guanylyl cyclise (sGC) is stimulated resulting in enhanced synthesis of cyclic guanosine monophosphate (GMP).

The vascular endothelium has many functions and, accordingly, endothelial dysfunction is responsible for numerous health problems including atherosclerosis, high blood pressure, sepsis, thrombosis, vasculitis, and bleeding, among others. One of the most important functions of the endothelium is to secrete nitric oxide (NO), a relatively unstable diatomic free radical, which can be synthesized by a broad range of organisms. This molecule has a role as a messenger in many biological processes in humans including involvement in the regulation of neurone communication, antimicrobial activity, ventilation, hormone secretion, inflammation and immune responses well as vascular tone [3]. Indeed, it is a potent vasodilator and levels are often reduced when endothelial function is impaired, making it a vascular risk factor and, in particular, together with dyslipidaemia, a risk factor for coronary disease. Nitric oxide was first described as an endothelium-derived vascular relaxant factor, but its role in vasodilation depends on an increase in the levels of cyclic guanosine monophosphate (cGMP) in smooth muscle cells (Figure 1). In this case, NO is synthesized from arginine by the enzyme nitric oxide synthase (NOS), which has been identified in neurons, endothelial cells, macrophages and hepatocytes in various different isoforms: inducible (iNOS) and constitutive (cNOS), which contains neuronal (nNOS), and endothelial (eNOS).

In the kidney, where the synthesis of arginine occurs mainly in the proximal tubules, cNOS has been found in the glomerules, vessels and tubular segments including the macula densa and inner medullary segments of the collecting duct system [4,5]. The iNOS isoform has been found in the smooth muscle cells of blood vessels, the distal end of the efferent arteriole and the medullary area of the ascending limb of the loop of Henle [6]. Cytokines that stimulate this inducible form (iNOS) have been found in cultures from proximal tubules, inner medullary segments of the collecting duct system and the mesangium [7].

Renal medullary blood flow varies in response to endothelium-dependent vasodilation and endothelial cells of the vasa recta renis are capable of producing NO, which can have an influence on transport within the collecting duct system. The inhibition of NO synthesis in the kidney may have numerous consequences for patients:

- Reduced glomerular blood flow, together with an increase in the vascular resistance of the afferent and efferent arterioles;
- Reduced ultrafiltration, renal blood flow and glomerular filtration rate (GFR);
- Decreased secretion of renin, a hormone involved in the sodium and water balance in the body;
- Reduced ability to excrete sodium under normal conditions;
- Increased blood pressure and deterioration in renal function;
- Lack of stimulation for Na^+^ and HCO_3_^−^ transport in the nephron proximal tubules mediated by cGMP;
- Production of oxygen reactive species;
- Production of nitric peroxide when exposed to superoxide anions.

In brief, it has relatively recently been discovered that NO is an important messenger molecule involved in numerous biological processes, and knowledge about its activity is essential to help us understand both pathogenic and therapeutic mechanisms in kidney disease.

### 2.1. Endothelial Dysfunction and Oxidative Stress in Kidney Disease

Several theories indicate that endothelial dysfunction is what predisposes individuals to rapid atherosclerosis that, in turn, is involved in the pathogenesis of high blood pressure and associated with renal function decline [8]. On the other hand, it has been suggested that a reduction in NOS activity or an increase in its degradation rate, due to oxidative stress, may cause endothelial damage [9].

Oxidative stress in patients who have undergone RTx is mainly due to endothelial dysfunction caused by the inhibition of NO synthesis. It should, however, be taken into account that the use of immunosuppressants also influences oxidative stress in patients after RTx, since cyclosporine and tacrolimus cause post-transplant hypertension, which weakens defenses against oxidative stress [10,11]. In the case of tracrolimus, it has been described how the immunosuppressant can cause inhibition of the activity and transcription of eNOS [12], leading to a reduction in NO levels. Reduced production of endothelial NO could also be a direct consequence of therapy with cyclosporine since it induces calciuria, and this would increase the risk of early atherothrombosis in patients after RTx [13].

It is very important to study the role of arginine in this type of patient, since NOS activity in kidney failure is determined by arginine concentration [14], the key role of the kidney in the production of the NO precursor making renal patients prone to NO deficiency.

### 2.2. Arginine-Nitric Oxide Metabolism

**Figure 2:**
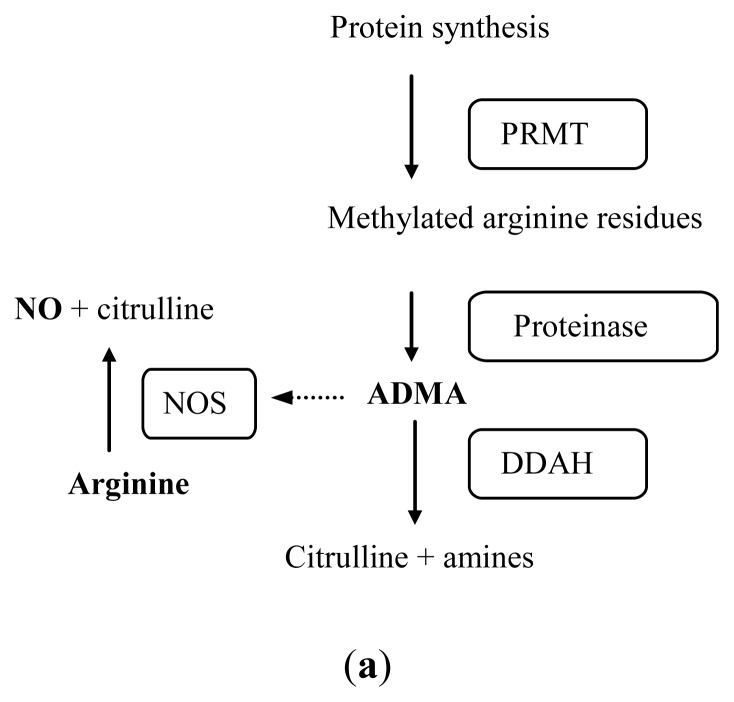

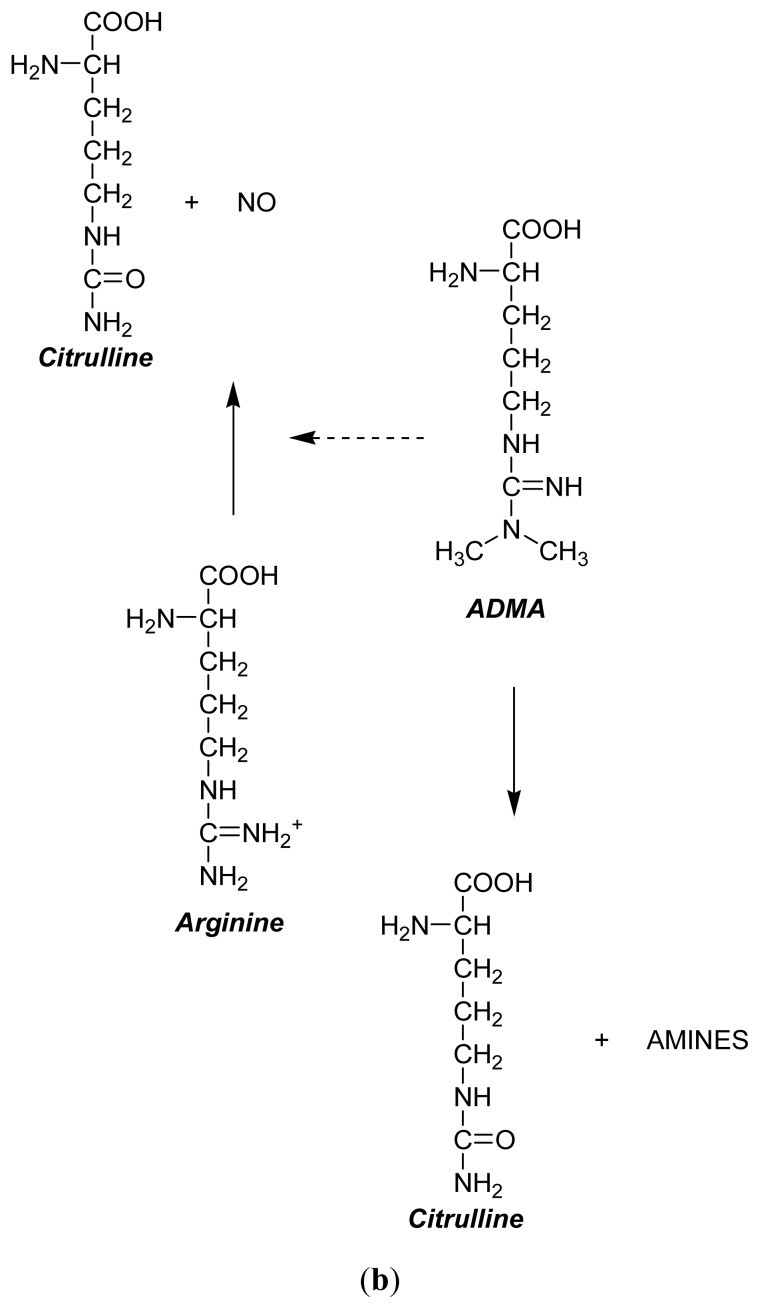
(**a**) Arginine-nitric oxide metabolic pathway. The abbreviations stand for the following compounds: nitric oxide synthase (NOS), asymmetric dimethylarginine (ADMA), nitric oxide (NO), protein arginine methyltransferase (PRMT) and dimethylarginine dimethylaminohydrolase (DDAH); (**b**) Molecular diagram illustrating the inhibitory role of asymmetric dimethylarginine (ADMA) on nitric oxide (NO) production.

l-arginine (Arg) can be broken down by several metabolic pathways: apart from its transformation into guanidinoacetate and creatine, it can be oxidized to l-citrulline (Citr) and NO by endothelial NOS (its main substrate) (Figure 2a). In line with this, acute or chronic administration of Arg increases the production of endothelial nitric oxide and improves endothelial function, decreasing the risk of atherothrombosis [15].

On the other hand, protein Arg residues can be methylated in various ways such that their subsequent proteolysis gives rise to three types of compounds: asymmetric dimethylarginine (ADMA), symmetric dimethylarginine (SDMA) and *N*-monomethyl-l-arginine (NMMA) (Figure 2b). Arg methylation is catalysed by type I and II protein arginine methyltransferase (PRMT), whose activity also depends on *S*-adenosylmethionine as a donor of methyl groups. Type I PRMT leads to ADMA, which is produced in all body cells and competes with Arg for the NOS isoforms, while type II PRMT gives raise to NMMA and SDMA, which does not directly affect NOS activity, but may compete with Arg in the transport system y+. Type I PRMT is expressed predominately in the endothelium and smooth muscle cells of the cardiovascular system [16]. Under oxidative stress, low-density lipoproteins (LDLs) and oxidized LDLs may increase the activity of the enzyme in endothelial cells, leading to an increase in the production of ADMA [17]. Unmetabolized ADMA is released to the blood stream by the membrane transport system y+. In addition, a small percentage (10%) of the clearance of ADMA occurs by urinary excretion. Further, although SDMA does not inhibit NO synthesis directly, it may compete with the cationic amino acid transporter of the membrane of the endothelial cells, accentuating the Arg deficiency and indirectly altering the level of NO production, it having been reported that SDMA itself significantly reduces Arg uptake in the loop of Henle of the rat nephrons [18]. For these reasons and the fact that SDMA is mainly eliminated through urine, many research groups are studying its correlation with renal function [19–21], with creatinine excretion [22], with inflammatory parameters [23], and with mechanisms that regulate blood pressure [24], and it is considered to be a marker for cardiovascular disease as others guanidinio compounds [25,26].

Most ADMA, but not SDMA, is degraded to Citr and dimethylamine (Figure 1a) by dimethylarginine dimethylaminohydrolase (DDAH), which is widely distributed throughout the body, particularly in the liver and kidney [27]. DDAH has two isoforms (I and II), DDAH I predominantly in tissue expressing nNOS and DDAH II in tissues containing eNOS [28]. The two isoforms are expressed differently depending on the tissue, and in this way regulate ADMA levels and hence NO synthesis [29]. For these reasons, reduced activity DDAH may be the main mechanism underlying an increase in ADMA levels.

## 3. ADMA as a Cardiovascular Risk Factor

**Figure 3:**
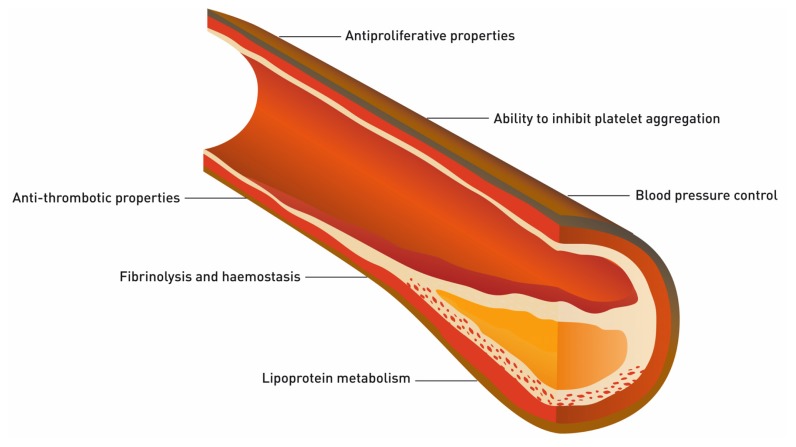
Representation of the main properties and functions of normal endothelium.

Several studies have found that the plasma levels of ADMA in healthy subjects with no apparent cardiovascular disease are related to age, blood pressure, insulin resistance, and carotid intima-media thickness [30,31]. These findings suggest that an increase in ADMA levels is a marker for arteriosclerotic changes. For this reason, ADMA levels have been used for assessing cardiovascular risk [32,33]. Further, it is known that ADMA is mainly absorbed by endothelial cells, extracellular levels being 5 to 10 times lower. This means that slight changes in plasma levels of ADMA are sufficient to significantly change intracellular ADMA levels and, thereby, modify NO production and contribute to the development of cardiovascular disease. Under pathological conditions, there may be a 3- to 9-fold increase in plasma levels of ADMA, and this would inhibit NO production by 30% to 70% [34]. This explains why this metabolite can be used as a cardiovascular marker, as a decrease in NO production by the some of the endothelium leads to adverse vascular effects (Figure 3) such as dysregulation of blood pressure, prevention of vasodilation, loss of antithrombotic activity, problems in homeostasis and fibrinolysis, and inhibition of platelet aggregation, among others [35,36]. Given this, the effect of ADMA on the Arg-NO pathway is of particular importance in conditions associated with vascular wall damage, such as hypercholesterolaemia, high blood pressure, smoking, diabetes and obesity.

Cardiovascular disease is responsible for 70% of deaths in patients with type 2 diabetes [37], but the levels of the conventional markers for cardiovascular disease are not sufficiently elevated to explain the excess cardiovascular risk in these patients. Given this, the plasma levels of ADMA have been analyzed in individuals with glucose intolerance [38], insulin resistance [39] and diabetes [40], elevated levels of the metabolite being associated with reduced DDAH activity, thoughout a polymorphism of the *DDAH2* gene in case of type 2 diabetes [41]. Several authors have reported a direct relationship between high blood pressure and ADMA levels [42,43]. Further, the relationship between the two factors has already been demonstrated in healthy subjects, who were administered methylarginine in order to increase their ADMA concentration to higher than normal levels, and in patients with end-stage renal disease in whom elevated ADMA levels were directly associated with carotid atherosclerosis and cardiovascular mortality [44,45].

Elevated ADMA levels have also been linked to oxidative stress [46] and hypercholesterolaemia [47,48], through a reduction in DDAH activity. It has recently been confirmed that smoking increases the levels of ADMA and decreases those of Arg, and this may be explained by impairment of the y+ transport system by tobacco smoke [49].

ADMA levels are elevated in humans and other animals with heart failure, specifically in patients with dysfunction and left ventricular hypertrophy [50], and also in patients with chronic kidney failure [51,52]. More recently, reduced DDAH activity has also been observed in the coronary endothelium, smooth muscle and cardiac myocytes in an animal model of kidney failure [53]. Therefore, this metabolite could be used as a marker for kidney failure as well as for cardiovascular risk.

### Relationship between ADMA and Homocysteine (Role of the DDAH Enzyme)

Various studies have failed to find a relationship between the levels of cardiovascular marker homocysteine (Hcys) and ADMA [54], both of which are elevated in paediatric patients after RTx. It is well known that Hcys causes endothelial dysfunction, and this could be due to the formation of disulphide bonds and generation of hydrogen peroxide and superoxide anions, which would increase the oxidative degradation of NO [55]. Hcys may also decrease NO synthesis through the degradation of NOS. A recent study demonstrated that an increase in Hcys causes a decrease in the activity of DDAH, which is able to degrade ADMA [56]. The resulting accumulation of ADMA impairs NOS function leading to a reduction in NO synthesis and accelerates the progression of atherosclerosis [46].

DDAH has four cysteine residues, whose sulfhydryl groups are of great importance for it activity, agents such as *p*-chloromercuribenzoic acid and mercury chloride that block these sulfhydryl groups being potent inhibitors of the enzyme. Similarly, Hcys also has very reactive sulfhydryl groups that are responsible for the production of Hcys dimmers and the formation of covalent bonds between Hcys and proteins. Therefore, it is quite possible that Hcys forms disulphide bonds to the DDAH enzyme, blocking the binding of ADMA to the active site of the enzyme and, thereby, slowing the degradation of the dimethylarginine molecule. Stühlinger *et al*. [46] indicated that the bonds between the Hcys and DDAH modify the spatial configuration of the enzyme, inhibiting its activity.

Further, the high reactivity of Hcys may cause the degradation of proteins, destabilizing their structure and increasing the level of oxidative stress. In this way, methylated arginine residues arising from the proteins are released giving rise to ADMA molecules. The increase in the plasma level of this methylated arginine is a better cardiovascular marker than Hcys: some authors have not found Hcys to be associated with cardiovascular events or carotid intima-media thickness in renal patients [57]; while, in contrast, analyzing the relationship between levels of ADMA and the survival of patients with chronic kidney disease using multivariate Cox models, elevated levels were found to be significantly associated with cardiovascular-related mortality [58].

Other studies have suggested that elevated levels of oxidized LDLs and lysophosphatidylcholine, their major component, lead to a reduction in DDAH activity thus increasing ADMA levels [59,60]. On the other hand, if LDL cholesterol levels are normal, this inhibitory mechanism can be ruled out as an explanation of the elevated ADMA levels in patients with renal dysfunction.

The fact that ADMA is correlated with few other variables, and in particular that it is not correlated with Hcys, reinforces the theory that ADMA is an independent cardiovascular biomarker [61]. In fact, it has been confirmed that the predictive cardiovascular value of ADMA is similar to that of B-type natriuretic peptide (a biomarker of heart failure) and C reactive protein (CRP, an inflammatory mediator). There are even predictive models based on these three markers, which provide greater predictive value for potential adverse cardiovascular events [62]. Higher concentrations of ADMA have also been found to be associated with a reduction in the urinary excretion of creatinine, and this illustrates the sense in which ADMA is a biomarker for kidney disease, levels being directly related to renal function [30,63].

Arginine acts as a natural precursor for NO production in the endothelium and other types of tissue. Accordingly, it would be interesting to compare the ratio between Arg and ADMA in patients with renal disease and a control group, as this value may indicate a potential problem with NO production. Some authors have already observed a reduction in the Arg/ADMA ratio in patients with renal disease, and this was associated with clinical manifestations of atherosclerosis and, accordingly, also with the regulation of processes such as blood pressure control, vasodilation, antithrombotic activity, homeostasis and fibrinolysis [64,65]. This ratio is of great importance for the absorption of Arg by cells and subsequent NO production, not only in renal tubular epithelial cells but also in endothelial cells.

## 4. Asymmetric Dimethylarginine in Kidney Disease

It has been observed that endothelial NO production is reduced in renal patients, in part due to limited synthesis of the substrate Arg but also because the levels of ADMA, a competitive inhibitor of NOS, are elevated [15]. These factors contribute to endothelial dysfunction, cardiovascular risk and the progression of renal damage.

ADMA accumulates in patients with chronic renal failure [64,66], inhibiting the activity of NOS, which may explain, at least to some extent, the high blood pressure and endothelial dysfunction seen in these patients. There have also been other observations that suggest the great biological importance of the activity of ADMA as a cause of endothelial dysfunction, reducing NO production via decreased eNOS phosphorylation [67,68]. In a recent review of the role of ADMA in chronic kidney disease [65], four different main mechanisms were identified to explain the accumulation of ADMA in this type of patient, and this analysis may be extrapolated to RTx [69]:

- Higher levels of protein methylation;
- Increased rate of protein turnover;
- Impaired activity of DDAH, which degrades ADMA;
- Impaired renal excretion.

It is worth noting that ADMA levels are elevated in patients with early phases of renal disease, even before glomerular filtration is significantly reduced [63]. This suggests that only a very small fraction of circulating ADMA is excreted through urine, although it is negatively correlated with GFR [32]. The importance of high levels of methylated arginine in renal disease is evidenced by the fact that patients have been diagnosed with chronic kidney disease despite having normal blood creatinine levels, and therefore GFR was not measured [70]. Some studies found lower DDAH activity in an animal model with kidney failure, showing increased levels of ADMA [53,71], these depending more on the activity of the enzyme that degrades it than on the GFR.

In some studies, elevated ADMA levels have not been found to be directly related to cardiovascular events either in the general population or in renal patients. This can be attributed to a state of malnutrition or an increase in inflammation together with oxidative stress [72]. In general, however, renal patients do have higher ADMA levels than control groups; accordingly, NO production is inhibited, contributing to endothelial dysfunction and cardiovascular risk in renal patients and limiting survival, given the association with heart disease [73,74]. Table 1 summarizes the values of Arg, ADMA and SDMA measured in control subjects and/or renal patients from various studies in recent years.

In young people, the metabolic pathway involving ADMA has been little studied despite evidence that levels of this molecule are clinically relevant [87]. Among the few studies reported to date, elevated levels of ADMA have been detected in children and adolescents with high blood pressure [42], and its levels have started to be investigated in children with chronic renal failure [24,88], and in children and adolescents with hypercholesterolaemia [89], diabetes [90] and phenylketonuria [91]. Table 2 summarizes reported measurements of ADMA in the paediatric population: levels are higher than in adults due to the immaturity of the enzyme system, affecting DDAH activity in children and adolescents.

### 4.1. Asymmetric Dimethylarginine and Proteinuria

Proteinuria and also microalbuminaemia are potent and independent markers of cardiovascular risk, and there is evidence of their close relationship with endothelial dysfunction and inhibition of NO production [93]. Given that ADMA has also been associated with endothelial dysfunction in animal models and patients with chronic kidney disease [94], it is conceivable that the development of proteinuria may have a role in the accumulation of ADMA via the overexpression of PRMT that facilitates the synthesis of ADMA [95]. In line with this, positive correlations have been observed between ADMA and proteinuria [96] and, in an animal model, some researchers have observed a decrease in the activity of the DDAH enzyme [97], which is abundantly expressed in the renal tubular cells.

### 4.2. Asymmetric Dimethlylarginine after Renal Transplantation

So far, there have been very few studies of ADMA levels in patients undergoing RTx. This is despite the fact that, given the association with cardiovascular risk, it is a metabolite of great interest in this group of patients. Yilmaz *et al*. [98] reported that, during their study follow-up period of four weeks, RTx greatly decreased ADMA levels, compared to before transplantation. Other studies have also highlighted that ADMA levels decrease during the first month after transplantation, but they remain elevated compared to a control population [85]. This fall results in an increase in NO synthesis and associated improvement in endothelial function, NO synthesis being closely associated with atherosclerosis in chronic kidney disease [99]. The fact that patients start immunosuppressive therapy does not seem to affect the improvement in endothelial function. This study also indicated that the levels of adiponectin, inflammatory and anti-atherogenic hormone are not correlated with the levels of ADMA or CRP before and after RTx, despite all the values corresponding to these variables having decreased after transplantation. Thus, as well as poor renal function, other factors may contribute to increasing levels of adiponectin in patients with chronic kidney disease. Further, the decrease in the inflammatory state after RTx may mask a correlation between GFR and adiponectin values.

The levels of the metabolites considered in this review must be analyzed taking into account that if the conversion of Arg into guanidinoacetate is impaired, the accumulation of Arg and its asymmetric derivatives may compromise NOS function and, in turn, have a negative effect on endothelial function; this would enhance the cardiovascular risk inherent to the use of immunosuppressive agents, increasing mortality and morbidity in these patients.

The mechanisms by which plasma ADMA levels may increase include an increase in the methylation of proteins and/or in proteolysis, and impairment of renal excretion of ADMA and/or of DDAH activity. In a patient population, it is relatively important to assess whether methylation activity is significantly enhanced (*S*-adenosylmethionine/*S*-adenosylhomocysteine ratio) in order to relate this to an increased methylation of proteins. In a recent study, we observed a significant increase in ADMA levels and a decrease in methylation activity in transplant recipients compared to a control population [92]. Other authors have suggested that obesity after RTx may be directly associated with elevated ADMA levels and a decrease in adiponectin, increasing cardiovascular risk [100,101]. Moreover, ADMA was found to be a significant risk factor for graft failure, so these plasma levels could predict morbidity, mortality and the deterioration of graft function RTx [102].

## 5. Treatments to Reduce ADMA Levels

Oxidative stress may increase the synthesis of ADMA, by stimulating the expression of methyltransferases in the body or reducing DDAH activity. Specific ADMA-lowering therapies are not yet available, but different dialysis models have different clearance capability on plasma ADMA [103], even angiotensin converting enzyme inhibitors and angiotensin receptor blockers may help to improve endothelial function and to prevent ADMA-induced pathology [104]. On the other hand, treatment with antioxidants of cells with very elevated intracellular ADMA levels restores the activity of DDAH. Given this, several studies have been initiated focusing on the use of antioxidants, such as tea and vegetable consumption [105], to re-establish endothelial production of NO.

One of these treatments includes the use of vitamin E that reduces lipid peroxidation and improves NO production, inhibiting the production of free radicals. Saran *et al*. [106] observed a 23% reduction in ADMA levels in patients with non-diabetic chronic kidney disease, after a period of eight weeks on supplementation with vitamin E. This confirms the success of this strategy of using antioxidants to reduce ADMA levels, increasing DDAH activity and reducing the levels of free radicals. The antioxidant power of vitamin E seems not to be strong enough to change the levels of Hcys and CRP, at least not with the doses and duration of treatment in the aforementioned study. Further, vitamin E hardly reduces ADMA levels at all in patients with high cardiovascular risk but, given its antioxidant nature, does normalize the levels in patients with chronic kidney disease and hyperhomocysteinaemia, in which there is elevated oxidative stress [107].

Impaired renal function and the presence of proteinuria or microalbuminaemia are commonly associated with endothelial dysfunction in patients with chronic kidney disease, in whom levels of ADMA are elevated and positively correlated with the reduction in renal function. For this reason, Teplan *et al*. [108] explored treating obese patients with chronic kidney disease with a low protein diet supplemented with keto-amino acids to maintain the nitrogen balance and prevent malnutrition. This type of treatment achieved a reduction in proteinuria, stabilization of renal function and a decrease in ADMA levels in these patients, methylarginine mainly coming from the proteolysis of proteins. Other studies suggest that a soy protein-rich diet can contribute to improving endothelial function by normalizing the Arg/ADMA ratio in kidney transplant recipients [109] using isoflavones, this treatment being easy to implement given that it just involves consumption soy protein in place of some animal protein.

New therapeutic approaches are currently being developed, in particular, based on the administration of intravenous *N*-acetylcysteine. This treatment not only lowers Hcys levels, but also reduces blood pressure and improves endothelial function [110], as well as decreasing ADMA levels in patients on haemodialysis [111]. *N*-acetylcytesine breaks disulphide bonds; the reason why this thyol has a direct antioxidant effect, decreasing the levels of oxygen reactive species and increasing the bioavailability of DDAH, is that it releases the enzyme from any molecule binding to it, such as Hcys, thus re-establishing normal enzymatic activity. In this way, it is possible to decrease levels of ADMA, as this molecule will be degraded to amine groups and Citr.

### 5.1. Statins

Given the high cardiovascular risk associated with elevated levels of ADMA and Hcys, it is logical to consider possibility of treating such patients with statins: in the long term, these agents having been found to improve the oxidative status of adult renal transplant patients and even increase their levels of HDL cholesterol, which has great antioxidant properties [112].

It is known that statins significantly reduce the number of episodes of acute transplant rejection, while improving graft survival and renal function. These effects are correlated with a decrease in the levels of triglycerides and a blocking of the synthesis of cholesterol and non-steroidal compounds derived from mevalonate, an early precursor of cholesterol. Further, statins may have an additional immunosuppressive effect through the inhibition of interleukin-6, in this way altering the initial stages of T-cell activation and, thereby, interrupting cellular rejection. This effect is also associated with a decrease in the levels of triglycerides mediated by interleukin-6 [113].

Initial studies in patients with chronic renal insufficiency have indicated that treatment with statins does not reduce ADMA levels to the normal range [107,114], although they do have a cardiovascular protective effect. While some studies have associated ADMA with LDL cholesterol [59,60], it seems that only certain types of statins reduce plasma levels of ADMA, since the vascular effects of these agents depend on the expression of the regulatory gene NOS [35]. Specifically, it has been reported that rosuvastatin contributes to reducing ADMA levels in children [47].

On the other hand, there are various concerns with respect to the use of statins given their most common adverse effects including fatigue, gastrointestinal problems including constipation and cramps. Accordingly, alternatives have started to be tested such as the effect on endothelial function of kidney transplanted patients of the consumption of fish rich in long-chain omega-3 fatty acids, which may also have a protective effect against the nephrotoxicity of some immunosuppressive agents [115].

While the dose-response relationship for omega-3 fatty acid supplementation remains to be clarified, it has already been demonstrated that this treatment is not associated with Hcys levels in patients with chronic kidney disease [116]. Similarly, the administration of statins to kidney transplant patients did not have an impact on plasma levels of Hcys, although it does manage to reduce cardiovascular risk by decreasing the levels of total and LDL cholesterol and triglycerides, without affecting the levels of HDL cholesterol [117]. For this reason, in the case of children and adolescents with renal insufficiency or those subject to transplantation, there are more doubts over the usefulness of statin treatment if the levels of triglycerides, total cholesterol and its subfractions are within normal ranges. In particular, if these patients have hyperhomocysteinaemia, the treatment would not reduce Hcys levels given that there is no association between the levels of Hcys and statins.

### 5.2. Arginine

In recent years, it has been demonstrated that Arg supplementation restores NO production, improves renal function and reduces inflammation in kidney transplant patients [118]. A key objective in renal patients is to decrease ADMA levels [119]. For this reason, arginine supplementation has been used, reversing ADMA-induced inhibition of NO synthesis. According to several studies, arginine also improves endothelial dysfunction in patients on haemodialysis by regulating ADMA levels [120]; that is, the inhibitory action of ADMA on NOS is reverted by arginine loading.

Arginine supplementation may increase the production of guanidinoacetate and creatine, through the conversion of *S*-adenosylmethionine to *S*-adenosylhomocysteine and Hcys, which may themselves have adverse effects on endothelial function [121]. In kidney transplant recipients, however, in whom there is already endothelial dysfunction for various reasons, supplementation with arginine contributes to improving renal function, normalizing the Arg/ADMA ratio, increasing NO production, and eliminating nitrites and nitrates, which improves endothelial function [122]. It has even been demonstrated that the intake of Citr may have similar effects since it is converted into Arg, with no adverse effects and with a good tolerance [123].

On the other hand, in patients with high plasma Arg and Citr levels, the efficacy of supplementation of this amino acid has been questioned due to its potential adverse effects. It should also be taken into account that Arg is an acidifying agent that may alter the acid-base equilibrium in patients with reduced GFRs; accordingly, in such cases, it should be administered in the form of arginine salts other than chlorates. This may explain why data on the effects of the arginine supplementation are inconsistent, some studies having found no significant beneficial effects on vascular function and others finding no differences in endothelial NO synthesis with respect to placebo [32].

## 6. Conclusions

Kidney disease is directly associated with elevated levels of the cardiovascular marker ADMA and a significant reduction in the Arg/ADMA ratio, such that NO synthesis is inhibited, worsening renal dysfunction and increasing cardiovascular risk.

The lack of correlation between ADMA and other variables strengthens the theory that it is an independent marker of cardiovascular risk. It has also been observed that ADMA levels tend to be higher in children, due to immaturity of the enzyme system, affecting DDAH activity.

Treatments to reduce ADMA levels may be useful in patients with kidney disease, but decisions should be taken on a case-by-case basis, including a complete analysis of the corresponding metabolic pathways and lipid profiles.

## Figures and Tables

**Table 1:** Concentrations of Arg and its methylated metabolites in plasma of healthy human subjects and patients with renal failure.

	Subjects	Age (Years)	ADMA (μM)	SDMA (μM)	Arg (μM)	Arg/ADMA	Method
Marescau *et al.* 1997 [75]	Controls	23–86	0.41 ± 0.09	0.38 ± 0.10	110 ± 24		LC-Fluorescence
	CKD: CC > 40	23–86	0.60 ± 0.10	0.83 ± 0.22	111 ± 24		LC-Fluorescence
	CKD: CC = 40–20	23–86	0.72 ± 0.22	1.41 ± 0.63	119 ± 33		LC-Fluorescence
	CKD: CC = 20–10	23–86	0.84 ± 0.15	2.24 ± 0.74	122 ± 36		LC-Fluorescence
	CKD: CC < 10	23–86	0.80 ± 0.14	3.17 ± 1.05	129 ± 37		LC-Fluorescence
Pi *et al*. 2000 [76]	Controls	23–35	0.30 ± 0.05	0.34 ± 0.06	60.7 ± 19.0		LC-Fluorescence
Tsikas *et al*. 2003 [77]	Controls	35.6 ± 11.4	0.39 ± 0.06				GC-MS/MS
Martens-Lobenhoffer *et al.* 2004 [78]	Controls	20–56	0.36 ± 0.07	0.46 ± 0.09	63.9 ± 23.9		LC-MS/MS
	CKD	36–78	0.67 ± 0.13	3.16 ± 0.91	48.1 ± 18.5		LC-MS/MS
Schwedhelm *et al*. 2005 [79]	Controls	>18	0.55 ± 0.14	0.69 ± 0.23	65.6 ± 23.4	132 ± 55	LC-MS/MS
Martens-Lobenhoffer *et al.* 2006 [80]	Controls	22–32	0.37 ± 0.06	0.45 ± 0.06	60.6 ± 18.3		LC-MS/MS
Wilcken *et al.* 2006 [81]	Controls	34.6 ± 11.7	0.49 ± 0.07	0.40 ± 0.07	87.9 ± 19.5	181.9 ± 56.1	LC-Fluorescence
	CBS	34.2 ± 12.6	0.55 ± 0.08	0.39 ± 0.09	73.5 ± 18.8	132.9 ± 24.7	LC-Fluorescence
Bishop *et al*. 2007 [82]	Controls	>18	0.66 ± 0.12		87 ± 35	142 ± 81	LC-MS/MS
Schwedhelm *et al.* 2007 [83]	Controls	>18	0.46 ± 0.09	0.37 ± 0.07	74 ± 19	166 ± 50	LC-MS/MS
Weaving *et al*. 2008 [84]	Controls	20.9 ± 2.5	0.40 ± 0.14	0.47 ± 0.06	162 ± 76		SPE-MS/MS
Zhang *et al*. 2009 [85]	Controls	46.1 ± 13.2	0.49 ± 0.12	0.24 ± 0.08			LC-Fluorescence
	CKD	45.7 ± 14.2	2.36 ± 0.89	0.48 ± 0.11			LC-Fluorescence
	RTx	45.7 ± 14.2	0.70 ± 0.24	0.26 ± 0.07			LC-Fluorescence
El-Khoury *et al.* 2012 [86]	Controls	19–64	0.36–0.67	0.32–0.65	53.1–129.7		LC-MS/MS

CBS: Cystathionine β-synthase deficiency; CKD: Chronic kidney disease; RTx: Renal transplant; CC: Creatinine clearance (mL/min).

**Table 2:** Concentrations of Arg and its methylated metabolites in plasma of children and adolescents.

	Subjects	Age (Years)	ADMA (μM)	SDMA (μM)	Arg (μM)	Arg/ADMA	Method
Wang *et al.* 2007 [88]	Controls	12.6 ± 1.0	0.78 ± 0.16	0.71 ± 0.23	65.3 ± 21.3		LC-MS/MS
	CKD	11.3 ± 4.7	1.10 ± 0.35	2.06 ± 1.11	57.9 ± 22.1		LC-MS/MS
Brooks *et al.* 2009 [24]	Controls	11.3 ± 4.7	0.8 ± 0.2	0.7 ± 0.2	65.3 ± 21.3	86.8 ± 30.6	LC-MS/MS
	CKD	12.6 ± 1.0	1.1 ± 0.3	2.1 ± 1.1	57.9 ± 22.1	62.4 ± 27.7	LC-MS/MS
Andrade *et al.* 2011 [92]	Controls	7–18	0.41–0.96		56.4–125.4	83.0–218.5	ELISA
	RTx	7–18	0.67–1.28		52.6–140.3	55.4–177.2	ELISA
Huemer *et al*. 2012 [91]	Controls	11.6 ± 3.7	0.64 ± 0.66		59 ± 59	86 ± 91	ELISA

CKD: Chronic kidney disease; RTx: Renal transplant.

## Abbreviations

ADMA: asymmetric dimethylarginine
Arg: l-arginine
cGMP: cyclic guanosine monophosphate
Citr: citrulline
cNOS: constitutive nitric oxide synthase
CRP: C reactive protein
DDAH: dimethylarginine dimethylaminohydrolase
eNOS: endothelial nitric oxide synthase
GFR: glomerular filtration rate
Hcys: homocysteine
iNOS: inducible oxide nitric synthase
LDL: low density lipoproteins
NMMA: *N*-monomethyl-l-arginine
nNOS: neuronal oxide nitric synthase
NO: nitric oxide
NOS: nitric oxide synthase
PRMT: protein arginine methyltransferase
RTx: renal transplantation
SDMA: symmetric dimethylarginine
